# Raltegravir switch improves hepatitis C transaminitis in HIV-1 and hepatitis C (HCV) co-infected individuals

**DOI:** 10.1186/1742-4690-9-S1-O7

**Published:** 2012-05-25

**Authors:** Muge Cevik, Gurmit Singh, Laura Dickinson, Andrew Scourfield, Marta Boffito, Mark Nelson

**Affiliations:** 1 Chelsea and Westminster Hospital, London, UK

## Introduction

HCV is one of the most relevant co-morbidities seen in HIV-infected individuals as evidenced by the negative impact that HIV exerts on the course of HCV infection. Despite remarkable results on HIV infection alone, the impact of highly active antiretroviral therapy (HAART) on liver disease in co-infection remains unknown. We sought to explore the impact of Raltegravir (RAL) on amino transferase (ALT) in HIV/HCV co-infected individuals.

## Methods

HIV-infected individuals co-infected with HCV within the last 5 years receiving non-integrase inhibitor containing HAART with a subsequent switch to RAL-containing HAART were identified from a retrospectively maintained outpatient database. Patient demographics were extracted. Biochemical, virological and immunological parameters were collated and individuals received pegylated interferon with ribavirin were excluded. ALT levels at switch and post switch were compared using Kruskal-Wallis test. Spearman’s Rank correlation was used to assess the relationship between ALT and HCV-RNA.

## Results

Twenty seven HIV-HCV co-infected individuals were identified between January 2007 and January 2012 and seven individuals were excluded. Median age was 44 years (range: 31-68). Five had acute and fifteen had chronic HCV infection during the switch. Twenty (100%) had HIV-RNA-1.

## Conclusion

In our study, RAL had a favourable effect on the liver up to 24 weeks after switch in HIV/HCV infected individuals.

